# Comparison Between the Likert Scale and the Visual Analog Scale for the 3D Esthetic Assessment of Primary Cleft Lip Repair: A Prospective Study

**DOI:** 10.1155/ijod/3592943

**Published:** 2026-10-06

**Authors:** Edoardo Staderini, Federica Guglielmi, Carmen Chiara Nacca, Licia Leccese, Antonino Fiorino, Massimo Cordaro, Mario Zama

**Affiliations:** ^1^ Department of Head and Neck and Sense Organs A. Gemelli University Policlinic IRCCS Catholic University of Sacred Heart Rome 00168 Italy unicatt.it; ^2^ Postgraduate School of Orthodontics Catholic University of the Sacred Heart Rome 00168 Italy unicatt.it; ^3^ Postgraduate School of Pediatric Dentistry Catholic University of the Sacred Heart Rome 00168 Italy unicatt.it; ^4^ School of Dentistry Catholic University of the Sacred Heart Largo Agostino Gemelli 8 Rome 00168 Italy unicatt.it; ^5^ Department of Neuroscience Reproductive Sciences and Dentistry Federico II University of Naples Naples Italy unina.it; ^6^ Department of Plastic and Maxillofacial Surgery Children’s Hospital Bambino Gesù IRCCS Rome Italy ospedalebambinogesu.it

**Keywords:** lay observers, Likert, unilateral cleft lip and palate, 3D stereophotogrammetric evaluation, VAS

## Abstract

**Objectives:**

To compare the agreement, reliability and convergent validity of the five‐point ordinal Likert scale and a 100 mm visual analog scale (VAS) for three‐dimensional (3D) esthetic assessment of primary unilateral cleft lip repair using a counterbalanced crossover design.

**Materials and Methods:**

Fifty lay observers (25 males, 25 females) evaluated 3D stereophotogrammetric facial models of 10 children with nonsyndromic unilateral cleft lip and palate at three time points: preoperative (T0), 1‐month postoperative (T1), and 6‐months postoperative (T2). Nine morphological items were assessed using both the five‐point Likert scale and a 100 mm VAS. A counterbalanced crossover design with a 2‐week washout period and inversion of scale order was implemented to minimize recall and order effects. Pairwise agreement analyses between the categorized VAS and the five‐point Likert scale (interscale, test–retest, presentation‐mode, and longitudinal agreement) were assessed using Cohen’s kappa (*κ*). Overall inter‐rater reliability was evaluated using the intraclass correlation coefficient (ICC), while convergent validity between the categorized VAS and the five‐point Likert scale was assessed using Spearman’s rank correlation coefficient.

**Results:**

Pairwise agreement between the categorized VAS and the five‐point Likert scale ranged from *κ* = 0.54 to *κ* = 0.80, indicating moderate‐to‐substantial agreement. Overall inter‐rater reliability was excellent for both instruments (ICC = 0.849–0.982). Test–retest agreement was slight to fair (*κ* = −0.05 to 0.49), while agreement between full‐face and cropped image presentation was generally limited (*κ* = −0.01 to 0.43). Longitudinal agreement across surgical stages was uniformly slight (*κ* = −0.05 to 0.18), reflecting expected changes in postoperative morphology rather than measurement instability. Convergent validity between the categorized VAS and the five‐point Likert scale was excellent (Spearman’s *ρ* = 0.948–0.974; all *p*  < 0.001). Subgroup analyses by evaluator sex and cleft index revealed no consistent patterns.

**Conclusions:**

Both the five‐point Likert scale and the VAS demonstrated excellent overall inter‐rater reliability and excellent convergent validity. However, because agreement analyses required categorization of the continuous VAS, the present findings do not establish whether preservation of its continuous measurement properties would provide additional discriminatory capability.

**Clinical Relevance:**

For 3D esthetic assessment of primary unilateral cleft lip repair, both the five‐point Likert scale and the VAS provide reliable group‐level evaluations. Standardized image presentation protocols and the use of multiple evaluators are recommended to improve the consistency of esthetic auditing.

## 1. Introduction

Unilateral cleft lip and palate (UCLP) is a significant congenital craniofacial anomaly that requires complex, multidisciplinary management [1]. Primary surgical repair aims at restoring lip integrity and function, guiding maxillary growth and facilitating speech, and improving facial appearance [2]. The esthetic outcome of the nasolabial region is a critical determinant of a patient’s quality of life, as facial attractiveness profoundly influences the development of self‐esteem and personality from infancy to adulthood [3–5].

The assessment of surgical outcomes is notoriously subjective, often leading to discrepancies between the perceptions of healthcare professionals and laypeople. [4, 6] While two‐dimensional (2D) photography has historically been the standard, it lacks depth perception and is prone to perspective errors. [7, 8] Recent studies have demonstrated that three‐dimensional (3D) images provide a more accurate and reliable platform for analyzing nasolabial shape and symmetry compared to standard photographs. [9, 10] Furthermore, longitudinal studies have shown that tracking 3D nasolabial changes is essential to monitor the long‐term success of primary repairs and the potential need for revision surgery [11, 12].

A critical component of esthetic research is the choice of the rating tool, as different scales can yield varying levels of sensitivity and reliability [13–15].

The original Asher–McDade Scale is a photograph‐based assessment tool that uses standardized photographic reference anchors to evaluate nasolabial appearance in patients with cleft lip and palate. In the present study, these morphological assessment criteria were adapted into a five‐point Likert scale without the original photographic reference anchors. This approach preserved the anatomical domains of the original framework while allowing standardized ordinal assessment of 3D stereophotogrammetric models.

To preserve these well‐established anatomical assessment criteria while adapting them to a 3D environment, clinical researchers frequently employ a five‐point Likert scale derived from the original Asher–McDade morphological framework [14, 15]. The Likert scale is an ordinal response system designed to convert subjective qualitative perceptions into discrete numerical categories along a standardized spectrum.

The visual analog scale (VAS) has been proposed as an alternative rating system because its continuous measurement structure may provide greater theoretical measurement resolution than ordinal categorical scales [13, 16].

Originally validated for clinical pain intensity measurement, the VAS is a continuous line (typically 100 mm) representing a spectrum from “very poor” to “very good” appearance [8]. Unlike ordinal scales, the VAS allows for more granular data collection, as observers could mark any point along the continuum [9].

In cleft research, the VAS has been proposed as a potentially more sensitive instrument because its continuous measurement structure may facilitate the detection of subtle esthetic differences. However, whether this theoretical advantage translates into improved performance when evaluating 3D stereophotogrammetric models remains uncertain [11, 14, 17].

Because direct agreement analyses require harmonized rating categories, the present study was designed to compare agreement, reliability, and convergent validity between the two assessment systems rather than to determine whether preservation of the original continuous VAS provides superior discriminatory performance.

Previous comparisons performed in 2D imaging environments suggest that both instruments provide reliable esthetic assessments, although their relative advantages remain a matter of debate [2]. However, there is a paucity of literature comparing the reliability of VAS and Likert scale for the 3D esthetic assessment of primary surgical repair [18]. The transition from 2D photography to 3D stereophotogrammetry introduces additional layers of complexity; the added depth perception and the ability to rotate models can either enhance the precision of ordinal scoring or, conversely, favor the granularity of a continuous scale like the VAS [3, 7]. Specifically, previous research has highlighted that standardized rating scales can be significantly influenced by the viewing perspective, with the basal view providing critical diagnostic information on nasal symmetry and projection that is often missed in frontal assessments [19].

Despite the clinical relevance of both systems, there is no definitive consensus on which scale is most effective for evaluating 3D facial models of children with UCLP.

Moreover, it is important to underline that most existing studies comparing these scales refer to secondary surgical outcomes or end‐of‐growth surgical revisions.

The present study aimed to compare the agreement, reliability, and convergent validity of a five‐point Likert scale and a VAS for the 3D assessment of esthetic outcomes following primary cleft lip repair. The objective is to generate evidence to support future audits of cleft lip treatment outcomes [14].

The null hypothesis was that no significant differences would be observed between the five‐point Likert scale and the VAS with respect to agreement, overall inter‐rater reliability, convergent validity, and the ranking of esthetic outcomes in 3D models of patients with UCLP.

## 2. Materials and Methods

### 2.1. Study Design and Ethical Approval

This observational reliability study with a crossover design was conducted at the Institute of Dentistry of “Fondazione Policlinico Agostino Gemelli” Hospital in cooperation with Craniofacial Centre of the Children’s Hospital “Bambino Gesù”; the study protocol was independently reviewed and approved by the Ethics Committee on May 31, 2017, with the ID number 0027003/17. The present investigation was reported according to the Strengthening the Reporting of Observational Studies in Epidemiology (STROBE) statement [20] (Supporting Information 1: File “Strobe statement”). Data analysis and reporting were further supported by the OHStat guidelines to ensure methodological transparency in reporting measurement reliability [21].

Informed consent was obtained from all parents and legal guardians of the patients about the treatment plan, the participation in the study, and the use of 3D images of their children; the case study was conducted in compliance with the principles of the Declaration of Helsinki, and with the understanding and written consent of both parents (Supporting Information 2: File 2).

### 2.2. Participants

Ten pediatric patients with nonsyndromic UCLP (seven males, three females) were prospectively recruited from the Craniofacial Centre of the “Bambino Gesù” Children’s Hospital (Rome, Italy) between June 27 and September 22, 2017. A consecutive sampling approach was utilized, including subjects without syndromic conditions or severely compromised medical status. To maintain cohort homogeneity and prevent evaluation bias, infants with a history of prior orthopedic or surgical interventions in the oral or nasolabial regions were excluded. Although standard clinical guidelines often suggest a target weight of 6 kg or an age of 6 months for surgery [22], the participants in this study underwent primary repair at ~10 weeks of age, adhering to the center’s established protocol. The surgical procedure was standardized using Millard’s technique for the reconstruction of the lip and nasal soft tissues, with all operations performed by a single senior surgeon to eliminate interoperator variability [4].

Preoperative deformity severity was classified according to the unilateral cleft lip severity index (UCL‐CSI), which categorizes cleft severity into 4 grades based on lip height involvement and nostril width ratio (NWR) [23]. In the present study, subgroup analyses were conducted comparing patients with Cleft Index 2 (incomplete cleft extending > 50% of lip height; *n*: four patients) and Cleft Index 4 (severe complete cleft with NWR > 2; *n*: six patients) to evaluate whether baseline morphological severity influenced the lay observers’ esthetic perception.

### 2.3. Sample Size Rationale

No formal a priori sample size calculation was performed because the present investigation was designed as a methodological reliability study rather than as a clinical effectiveness trial. The number of patients was determined by the availability of complete prospective 3D stereophotogrammetric records acquired at all predefined follow‐up stages. To maximize the precision of the reliability analyses, the study included a large panel of 50 independent lay observers, each evaluating all patients using both rating systems (five‐point Likert scale and VAS), under two presentation modes, across two assessment sessions (test and retest), and at three surgical stages. Overall, across the three surgical time points (evaluating seven items at T0 and nine items at T1 and T2 under two presentation modes), each observer completed 2000 individual ratings (10 patients × 50 morphological assessments × 2 rating scales × 2 sessions), resulting in 100,000 individual ratings across the panel of 50 lay observers. Although only 10 patients were included, this fully crossed repeated‐measures design generated a large number of paired observations for evaluating agreement, reliability, and convergent validity between the two rating systems. The limited number of patients may affect the external generalizability of the findings but does not compromise the primary methodological objective of comparing the measurement properties of the two esthetic assessment instruments.

Furthermore, the robustness of these reliability estimates is strengthened by the large number of evaluators included in this study. The recruitment of 50 independent lay observers constitutes a sample size that is substantially larger than those typically reported in comparable literature. Previous validation studies evaluating cleft lip esthetic outcomes—using both the Asher‐McDade scale and the VAS—have traditionally relied on much smaller panels, frequently ranging from 6 to 15 raters, and rarely exceeding 40–45 lay judges. [3, 13, 24–26] Thus, the current panel size provides a robust basis for the agreement and consistency analyses.

### 2.4. 3D Image Acquisition

3D facial images were acquired from the enrolled patients at three predefined longitudinal time points: preoperative (T0), 1‐month postoperative (T1), and 6‐month postoperative (T2). In line with the methodology described by Staderini et al. [4] all images were acquired using the 3dMDface System (3dMD, Atlanta, GA). Patients were positioned with a neutral facial expression, eyes open, and teeth in light contact. The same standardized acquisition protocol was applied at all three time points to ensure consistency of image capture throughout the longitudinal evaluation [4, 27].

### 2.5. Esthetic Assessment and Rating Scales

The esthetic evaluation was performed using a questionnaire with two distinct scoring systems to compare their reliability in a 3D environment. The five‐point Likert scale, adapted from the anatomical assessment framework originally proposed by Asher‐McDade et al. [28] was selected as the ordinal rating instrument for the present study [14, 28] While the original scale uses a 1 (very good) to 5 (very poor) direction, for the purposes of this study, to facilitate direct comparison with the VAS, the direction of the ordinal scoring system was reversed so that higher scores consistently indicated better esthetic outcomes on both instruments [2]. Scores ranged from 1 (very poor appearance) to 5 (very good appearance) [13].

The VAS was selected because its continuous measurement structure allows observers to record esthetic judgments along a continuum rather than within predefined ordinal categories [13]. Raters independently assessed the nasolabial region of each 3D model using both rating systems. The VAS consisted of a 100 mm horizontal line anchored by “very poor appearance” (0 mm) and “very good appearance” (100 mm) [6]. For agreement analyses, VAS scores were subsequently categorized into five ordinal classes to enable direct comparison with the five‐point Likert scale, while the original continuous VAS was retained for descriptive analyses [6].

### 2.6. Esthetic Outcomes

The rating form was administered in Italian. The esthetic evaluation was based on nine distinct morphological items designed to capture the complexity of the surgical outcomes: [19]

• Nasal and Labial Symmetry: Nasal profile, labial profile, and overall nose symmetry.
• Nostril Morphology: Size, shape, and symmetry of the nostrils.
• Anatomic Landmarks: Symmetry of the columella (Q5) and continuity of the upper vermilion border (Q8).
• Clinical Outcome: Quality of wound healing (Q9).

### 2.7. Raters

A total of 50 lay observers (25 males and 25 females), aged between 20 and 25 years, were included. The inclusion criteria were: (1) undergraduate student status with no extensive professional knowledge of cleft lip and palate conditions; (2) normal visual abilities without the use of contact lenses; (3) no use of medications potentially affecting visual perception or attentional control and no history of past or present neurological disorders; (4) no authorship or participation in previous studies involving facial esthetic assessment; and (5) absence of familiarity or close personal relationships with patients affected by UCLP. All observers were blinded to the aim of the study, the study design, patient‐related information, and the timing of image acquisition (T0 vs. T1 vs. T2), in order to minimize expectancy and information bias.

### 2.8. Evaluation Procedure, Familiarization, and Bias Control

The esthetic assessment utilized a set of nine items (Q1–Q9), which were adapted from the clinical outcomes auditing framework originally proposed by Asher‐McDade et al. [28] and are detailed in Supporting Information 2: File 2. These criteria are well‐established in the literature, having been widely validated through major multicenter initiatives such as the Eurocleft and Americleft projects. The items were further refined for application in a 3D stereophotogrammetric environment, following the methodological validation patterns established in other studies [13]. The 3D models were displayed on a high‐resolution monitor in a randomized order using Viewbox 4 (dHAL Software, Kifissia, Greece), with the presentation sequence generated through a computer‐randomization program (http://randomization.com/). To ensure strict protocol adherence, all sessions were conducted in a supervised laboratory setting where investigators monitored the process and addressed any procedural queries during an initial instructional briefing. Prior to the formal assessment, raters underwent a standardized orientation session where they were familiarized with a digital rating form and provided with an explanatory legend and landmark‐based guidelines for the morphological analysis of the nine items. It should be noted that while items Q1 through Q7 (related to nasal and lip symmetry/form) were evaluated at all time points (T0, T1, T2), items Q8 (continuity of the upper vermilion border) and Q9 (wound healing) were specifically designed for the postoperative evaluation. Consequently, these two items were only administered for the 1‐month (T1) and 6‐month (T2) postoperative models, as they are not applicable to the preoperative deformity. During the evaluation, examiners were permitted to independently rotate and zoom the volumetric models to inspect anatomical depth and subtle nuances. However, they were strictly instructed to orient each 3D model into specific perspectives, including frontal, lateral, and neck hyperflexion views, prior to scoring each morphological item. The mandatory neck hyperflexion view was considered essential for standardized assessment of the basal nasal complex, as previous studies have shown that esthetic ratings may be influenced by the viewing perspective [19].

While no specific time limits were imposed for individual “one‐shot” judgments, the entire evaluation was typically completed within ~60 min. To minimize order effect bias, a counterbalanced crossover design was implemented:

• Session 1: Half of the raters evaluated the images using the Likert scale followed by the VAS, while the other half started with the VAS.
• Session 2: Performed after a 2‐week washout period to prevent recall bias, the order of the scales was inverted for each rater.

This inversion was implemented to reduce potential carryover and rater fatigue effects by balancing the order of scale administration between assessment sessions.

To enhance the validity of the data, several measures were implemented to control for systematic errors. Examiners were provided with an explanatory legend and were specifically cautioned against the reflexive use of the central score (3) on the Likert scale. Participants were instructed to use the full range of the Likert scale according to their esthetic judgment.

Several potential sources of bias inherent to observer‐based esthetic assessments were considered during study design. Although the relatively small number of 3D models may limit external generalizability, the use of standardized pre‐ and postoperative images for each participant enabled within‐subject comparisons, thereby reducing interindividual variability. To minimize information and detection bias, all raters were blinded to patient identity, clinical information, study aims, and surgical timing, and models were presented in a randomized order using anonymized codes. Nevertheless, the possibility of imperfect blinding to treatment stage cannot be entirely excluded, as postsurgical morphological features may be recognizable in some cases. Observer‐related biases, including recall, order, and fatigue effects, were addressed through a counterbalanced crossover design, a washout period between rating sessions, and the separation of scale administrations. In addition, a structured training session with standardized definitions and examples was conducted to reduce misclassification and central tendency bias associated with subjective esthetic ratings. Finally, the selection of lay observers reflects the intended focus on nonclinical esthetic perception; although this may limit generalizability to expert‐based evaluations, it reflects a methodological choice in agreement with the study objectives.

### 2.9. Statistical Analysis

All analyses were performed using Stata 19 (StataCorp, College Station, TX, USA). Descriptive statistics were calculated for each item and time point, reporting mean values for subgroup comparisons.

The VAS consisted of a 100 mm continuous scale but was digitally recorded on a 0–10 metric. To permit direct comparison with the five‐point Likert scale, VAS scores were categorized into five ordinal classes exclusively for agreement analyses as follows: ≤1.99 (category 1), 2.00–3.99 (category 2), 4.00–5.99 (category 3), 6.00–7.99 (category 4), and ≥8.00 (category 5). This categorization was performed solely to enable paired comparisons with the Likert scale and did not replace the continuous nature of the original VAS for descriptive analyses. Accordingly, the present study was not designed to determine whether preservation of the original continuous VAS would provide greater discriminatory performance than the harmonized ordinal comparison adopted for agreement analyses.

Agreement between paired measurements was quantified using Cohen’s kappa (*κ*). Cohen’s *κ* was applied exclusively to paired comparisons and was used to evaluate: (1) interscale agreement between the categorized VAS and the five‐point Likert scale; (2) test–retest agreement within each rating scale; (3) agreement between presentation modes (full‐face vs. cropped images); and (4) longitudinal agreement between surgical stages. Accordingly, Cohen’s *κ* was not used to estimate agreement among multiple observers.

Because both the categorized VAS and the Likert scale are ordinal variables, the use of weighted Cohen’s *κ* was considered during the statistical planning. However, unweighted Cohen’s *κ* was retained because the primary objective of the analysis was to evaluate exact categorical agreement between paired ratings, whereas overall inter‐rater reliability and convergent validity were additionally assessed using the intraclass correlation coefficient (ICC) and Spearman’s rank correlation coefficient, respectively. Agreement among the 50 lay observers was assessed separately using ICCs, which are more appropriate for evaluating reliability in studies involving multiple raters. ICC estimates were calculated for each rating scale, morphological item, presentation mode, and surgical stage. ICC estimates were calculated using a two‐way random‐effects model with absolute agreement and average measures.

For analyses requiring a single summary estimate, the average of the test and retest ratings was calculated and subsequently used for comparisons between presentation modes, longitudinal agreement analyses, and correlation analyses. Longitudinal *κ* coefficients were interpreted as agreement between ratings obtained at different clinical stages and not as statistical measures of responsiveness or sensitivity to clinical change. Therefore, low longitudinal agreement was interpreted in the context of expected postoperative morphological changes rather than as evidence of inadequate measurement performance.

Convergent validity between the categorized VAS and the five‐point Likert scale was assessed using Spearman’s rank correlation coefficient, calculated for each morphological item, presentation mode, and surgical stage using the average of the test and retest ratings.

Exploratory subgroup comparisons according to evaluator sex and cleft severity (Cleft Index) were performed descriptively. For each morphological item, rating scale, presentation mode, surgical stage, and evaluation session, mean scores and standard deviations were calculated separately for each subgroup. No inferential statistical tests were applied to these comparisons, and no *p*‐values were generated. This approach was adopted because these comparisons were not prespecified as primary or secondary hypotheses of the study, and the limited sample size within subgroups (particularly for cleft severity, where Cleft Index 2 included 4 patients and Cleft Index 4 included 6 patients) would have rendered formal inferential testing underpowered and potentially misleading. Under these conditions, descriptive reporting of mean scores was considered the most appropriate and transparent analytical strategy. These subgroup analyses were intended solely to provide a descriptive overview and should be interpreted as hypothesis‐generating rather than confirmatory. All statistical tests were two‐sided, and statistical significance was set at α = 0.05.

## 3. Results

Agreement analyses were performed at four levels: (1) interscale agreement between the categorized VAS and the five‐point Likert scale, (2) test–retest agreement within each scale, (3) agreement between full‐face and cropped presentation modes, and (4) longitudinal agreement across surgical stages (T0, T1, T2). In addition, multirater reliability among lay observers was assessed using ICC, and exploratory subgroup analyses were conducted by evaluator sex and cleft index.

### 3.1. Agreement, Reliability, and Convergent Validity Between the Categorized VAS and the Five‐Point Likert Scale (Tables 1–3)

**Table 1:** Interscale agreement between the categorized visual analog scale (VAS) and the five‐point Likert scale at Test (T) and Retest (RT) sessions.

Measurement		Interscale agreement					
		FF_Pre	FF_1 m	FF_6 m	Pre	1 m	6 m
Q1	T	0.59	0.60	0.59	0.64	0.67	0.74
	RT	0.71	0.62	0.68	0.59	0.70	0.60
Q2	T	0.67	0.65	0.56	0.59	0.63	0.65
	RT	0.74	0.59	0.73	0.71	0.60	0.63
Q3	T	0.76	0.64	0.67	0.68	0.64	0.69
	RT	0.63	0.63	0.74	0.64	0.61	0.63
Q4	T	0.75	0.68	0.73	0.72	0.65	0.70
	RT	0.68	0.61	0.67	0.62	0.56	0.67
Q5	T	0.75	0.65	0.64	0.73	0.67	−0.06^a^
	RT	0.75	0.64	0.64	0.67	0.70	0.67
Q6	T	0.80	0.62	0.68	0.67	0.63	0.71
	RT	0.74	0.66	0.68	0.70	0.66	0.70
Q7	T	0.73	0.57	0.57	0.68	0.64	0.65
	RT	0.64	0.67	0.70	0.59	0.66	0.69
Q8	T	—	0.63	0.54	—	0.66	0.65
	RT	—	0.64	0.65	—	0.60	0.66
Q9	T	—	0.69	0.56	—	0.63	0.67
	RT	—	0.57	0.61	—	0.63	0.68

*Note:* Interscale agreement was calculated between paired ratings obtained using the categorized VAS and the five‐point Likert scale from the same evaluator during the initial test session (T) and the retest session (RT). Intrarater test–retest agreement within each individual scale across the two sessions is reported separately in Table 4. Cohen’s kappa values shown in this table do not represent agreement among different evaluators. Assessment of consistency among the 50 lay observers and the degree of overlap between the Likert scale and the categorized VAS. Q1–Q9: Morphological items evaluated, including nasal symmetry, vermilion border continuity, and wound healing. FF/Pre/1 m/6 m: FF = full‐face presentation; Pre = preoperative; 1 m and 6 m = postoperative follow‐up evaluations. “Pre”, “1 m”, and “6 m” without FF indicate cropped image presentation.

Abbreviations: RT, retest session performed after a 2‐week washout period; T, test session.

^a^Negative *κ* values indicate agreement lower than expected by chance. Given their isolated occurrence in the interscale comparisons, these values should be interpreted cautiously and not as evidence of systematic disagreement between the two rating systems.

**Table 2:** Overall inter‐rater reliability (ICC) among lay observers.

Time point	VAS ICC (95% CI)	Likert ICC (95% CI)
Preoperative	0.976 (0.948–0.993)	0.982 (0.960–0.995)
1 month	0.887 (0.755–0.965)	0.902 (0.786–0.970)
6 months	0.849 (0.687–0.953)	0.859 (0.705–0.956)

*Note:* Unlike the Cohen’s *κ* coefficients reported in Table 1, which quantify agreement between paired ratings (interscale agreement between the categorized VAS and the five‐point Likert scale, or test–retest agreement within the same evaluator), the ICC presented in this table estimates the overall reliability of the ratings provided by the entire panel of lay observers. Overall agreement among lay observers evaluated using a two‐way random‐effects ICC with absolute agreement for the FF presentation mode. ICC values were calculated from the average Test–Retest scores for each evaluator at each surgical stage. ICC model: Two‐way random‐effects model, absolute agreement. Pre/1 m/6 m: Preoperative, 1‐month postoperative, and 6‐month postoperative evaluations. Average ICC: Reliability of the mean ratings across all lay observers. 95% CI: 95% confidence interval.

Abbreviations: FF, full‐face presentation mode; ICC, intraclass correlation coefficient.

**Table 3:** Convergent validity between the categorized visual analog scale (VAS) and the five‐point Likert scale.

Presentation mode	Preoperative	1 month	6 months
Full‐face (FF)	*ρ* = 0.958	*ρ* = 0.962	*ρ* = 0.948
Cropped	*ρ* = 0.958	*ρ* = 0.974	*ρ* = 0.962

*Note:* Spearman’s correlation was calculated to assess the convergent validity between the two rating systems while preserving their ordinal ranking. All correlations were statistically significant (*p* < 0.001), indicating a very strong monotonic association between the categorized VAS and the five‐point Likert scale across all presentation modes and surgical stages. Assessment of the monotonic association between the categorized VAS and the five‐point Likert scale using Spearman’s rank correlation coefficient (*ρ*). Correlations were calculated using the average of the Test and Retest scores for each evaluator. Cropped: Cropped image presentation. Pre/1 m/6 m: Preoperative, 1‐month postoperative, and 6‐month postoperative evaluations. *ρ*: Spearman’s rank correlation coefficient.

Abbreviation: FF, full‐face presentation.

**Table 4:** Test–retest agreement within each rating scale.

Scale		Test–retest agreement					
		FF_Pre	FF_1 m	FF_6 m	Pre	1 m	6 m
Q1	VAS	0.19	0.19	0.17	0.29	0.19	0.22
	LIKERT	0.28	0.14	0.25	0.29	0.26	0.27
Q2	VAS	0.34	0.15	0.21	0.32	0.24	0.31
	LIKERT	0.48	0.18	0.32	0.44	0.25	0.34
Q3	VAS	0.35	0.20	0.23	0.25	0.23	0.27
	LIKERT	0.49	0.20	0.26	0.37	0.26	0.27
Q4	VAS	0.34	0.24	0.20	0.36	0.23	0.21
	LIKERT	0.42	0.17	0.24	0.40	0.28	0.31
Q5	VAS	0.34	0.20	0.23	0.35	0.21	−0.05
	LIKERT	0.30	0.20	0.18	0.34	0.22	0.30
Q6	VAS	0.36	0.18	0.33	0.37	0.23	0.26
	LIKERT	0.35	0.20	0.25	0.42	0.19	0.26
Q7	VAS	0.32	0.15	0.23	0.31	0.19	0.27
	LIKERT	0.32	0.13	0.21	0.38	0.27	0.30
Q8	VAS	—	0.16	0.27	—	0.21	0.33
	LIKERT	—	0.09	0.30	—	0.25	0.40
Q9	VAS	—	0.12	0.21	—	0.25	0.28
	LIKERT	—	0.10	0.21	—	0.25	0.35

*Note:* Cohen’s *κ* coefficients were calculated by comparing repeated evaluations performed by the same evaluator. Lower agreement values reflect within‐observer variability over time and should not be interpreted as disagreement between different evaluators. Assessment of agreement between repeated evaluations (test and retest) performed by the same evaluator after a 2‐week washout period. VAS/Likert: Comparison between the 100 mm visual analog scale and the five‐point ordinal Likert scale.

#### 3.1.1. Pairwise Agreement Between the Categorized VAS and the Five‐Point Likert Scale (Table 1)

Interscale agreement between the categorized VAS and the five‐point Likert scale demonstrated consistently moderate‐to‐substantial concordance across all morphological items, surgical stages, presentation modes (full‐face and cropped images), and evaluation sessions (test and retest). Across Q1–Q9, Cohen’s kappa coefficients ranged from *κ* = 0.54 to *κ* = 0.80, corresponding to moderate‐to‐substantial agreement according to the Landis and Koch [29] classification (Table 1). The highest agreement was observed for Q6 at the preoperative stage using full‐face images during the test session (*κ* = 0.8024). The lowest positive coefficient was observed for Q8 at the 6‐month postoperative assessment using full‐face images during the test session (*κ* = 0.5389). A single negative kappa coefficient was observed for Q5 at the 6‐month postoperative assessment using cropped images during the test session (*κ* = − 0.06). This value indicates agreement lower than expected by chance and should be interpreted cautiously given its isolated occurrence within an otherwise consistently positive pattern of interscale agreement across all remaining comparisons.

Agreement estimates were comparable between the test and retest sessions and remained relatively stable across surgical stages and presentation modes, indicating consistent categorical classification by the two rating systems under the different evaluation conditions.

Overall, the categorized VAS and the five‐point Likert scale demonstrated a consistently high level of categorical agreement across all evaluated conditions, indicating substantial overlap between the two rating systems. Detailed kappa coefficients for each item, presentation mode, surgical stage, and evaluation session are reported in Table 1.

#### 3.1.2. Overall Inter‐Rater Reliability (Table 2)

To complement the pairwise agreement analyses reported in Table 1, overall inter‐rater reliability among the panel of lay observers was additionally assessed using the ICC. A two‐way random‐effects model with absolute agreement demonstrated excellent reliability for both rating systems at all surgical stages. Overall inter‐rater reliability was excellent for both rating systems throughout the study. Average‐measures ICC values ranged from 0.849 (95% CI 0.687–0.953) to 0.976 (95% CI 0.948–0.993) for the categorized VAS and from 0.859 (95% CI 0.705–0.956) to 0.982 (95% CI 0.960–0.995) for the five‐point Likert scale. Although slightly higher ICC values were observed at the preoperative evaluation, all estimates remained within the range generally interpreted as excellent reliability. Detailed ICC estimates are reported in Table 2.

#### 3.1.3. Convergent Validity Between the Categorized VAS and the Five‐Point Likert Scale (Table 3)

Convergent validity between the categorized VAS and the five‐point Likert scale was excellent. Spearman’s rank correlation coefficients ranged from *ρ* = 0.948 to *ρ* = 0.974 across all presentation modes and surgical stages (all *p*  < 0.001). For full‐face images, correlation coefficients were *ρ* = 0.958 at the preoperative assessment, *ρ* = 0.962 at 1 month, and *ρ* = 0.948 at 6 months. Similar findings were obtained for cropped images (*ρ* = 0.958, *ρ* = 0.974, and *ρ* = 0.962, respectively). These findings demonstrate an excellent monotonic association between the two rating systems and support the observation that both instruments rank postoperative esthetic outcomes in a highly comparable manner. Detailed correlation coefficients are presented in Table 3.

### 3.2. Intra‐Rater (Test–Retest) Reliability (Table 4)

Test–retest agreement was consistently lower than the interscale agreement observed between the categorized VAS and the five‐point Likert scale across all items and surgical stages.

For the VAS, kappa coefficients ranged from *κ* = − 0.05 to *κ* = 0.37. The highest VAS intrarater value was observed preoperatively for Q6 (*κ* = 0.3736), whereas the lowest coefficient occurred at 6 months for Q5 (*κ* = − 0.0522). Preoperative VAS agreement ranged from *κ* = 0.1904 to *κ* = 0.3736, while values at 1 month ranged from *κ* = 0.1532 to *κ* = 0.2762. At 6 months, coefficients ranged from *κ* = − 0.0522 to *κ* = 0.3328.

For the Likert scale, kappa coefficients ranged from *κ* = 0.09 to *κ* = 0.49. Overall, the Likert scale demonstrated slightly higher test–retest agreement than the categorized VAS across most items and evaluation conditions. However, test–retest agreement remained generally low for both instruments. The highest intrarater value was observed preoperatively for Q3 (*κ* = 0.4868), whereas the lowest coefficient was recorded at 1 month for Q8 (*κ* = 0.0944). Preoperative Likert agreement ranged from *κ* = 0.2791 to *κ* = 0.4868, while values at 1 month ranged from *κ* = 0.0944 to *κ* = 0.3000. At 6 months, coefficients ranged from *κ* = 0.2107 to *κ* = 0.3999.

Across all items and evaluation conditions, test–retest *κ* coefficients generally remained within the slight‐to‐fair agreement range according to the Landis and Koch [29] classification. No consistent trend toward higher or lower repeatability was observed across postoperative stages. Overall, test–retest agreement remained within the slight‐to‐fair range for both rating systems throughout the study period.

Detailed Cohen’s *κ* coefficients for each item, presentation mode, surgical stage, and evaluation session are reported in Table 4.

### 3.3. Agreement Between Presentation Modes (Full‐Face vs. Cropped Images) (Table 5)

Agreement between presentation modes (full‐face vs. cropped images), calculated using the average of test and retest ratings, was generally limited across all morphological items and postoperative stages.

**Table 5:** Agreement between presentation modes (full‐face vs. cropped images).

Question	VAS			LIKERT		
	FF_Pre vs. Pre	FF_1 m vs. 1 m	FF_6 m vs. 6 m	FF_Pre vs. Pre	FF_1 m vs. 1 m	FF_6 m vs. 6 m
Q1	0.29	0.18	0.23	0.30	0.24	0.31
Q2	0.37	0.24	0.25	0.42	0.33	0.28
Q3	0.36	0.24	0.26	0.43	0.18	0.26
Q4	0.38	0.20	0.23	0.40	0.22	0.35
Q5	0.33	0.26	0.01	0.38	0.30	0.28
Q6	0.38	0.28	0.22	0.35	0.27	0.30
Q7	0.36	0.26	0.26	0.33	0.28	0.30
Q8	—	0.19	0.29	—	0.24	0.31
Q9	—	0.23	0.24	—	0.29	0.28

*Note:* Assessment of agreement between full‐face and cropped image presentations using the average of test and retest ratings. Cohen’s *κ* coefficients describe the consistency of categorical classifications across the two presentation modes at each surgical stage. FF = full‐face image presentation. Cropped = image presentation limited to the perioral region. Cohen’s *κ* coefficients were calculated using the average of test and retest evaluations. Lower agreement values indicate that esthetic classifications varied according to image presentation rather than reflecting disagreement between evaluators.

For the categorized VAS, Cohen’s *κ* coefficients ranged from *κ* = − 0.01 to *κ* = 0.38. The highest coefficient was observed for Q6 at the preoperative evaluation (*κ* = 0.3795), whereas the lowest value occurred for Q5 at the 6‐month postoperative evaluation (*κ* = − 0.0123). Preoperative agreement ranged from *κ* = 0.2886 to *κ* = 0.3795, decreased at the 1‐month evaluation (*κ* = 0.1828–0.2762), and remained low at 6 months (*κ* = − 0.0123–0.2863).

For the five‐point Likert scale, Cohen’s *κ* coefficients ranged from *κ* = 0.18 to *κ* = 0.43. The highest agreement was observed for Q3 at the preoperative evaluation (*κ* = 0.4257), whereas the lowest coefficient was recorded for Q3 at the 1‐month evaluation (*κ* = 0.1772). Preoperative coefficients ranged from *κ* = 0.3021 to *κ* = 0.4257, while lower agreement values were generally observed at the postoperative evaluations.

According to the Landis and Koch [29] classification, most comparisons fell within the slight‐to‐fair agreement range, with only isolated coefficients approaching moderate agreement. Overall, these findings indicate that esthetic ratings were influenced by presentation mode, with full‐face and cropped images frequently producing different categorical classifications despite depicting the same patient at the same surgical stage.

Detailed Cohen’s *κ* coefficients for each item and surgical stage are presented in Table 5.

### 3.4. Longitudinal Agreement Across Surgical Stages (Table 6)

Agreement between surgical stages (T0 vs.T1, T1 vs. T2, and T0 vs. T2), calculated using the average of test and retest ratings, was consistently low across both the categorized VAS and the five‐point Likert scale.

**Table 6:** Longitudinal agreement across surgical stages.

Question	VAS			LIKERT		
	T0 vs. T1	T1 vs. T2	T0 vs. T2	T0 vs. T1	T1 vs. T2	T0 vs.T2
Q1	0.10	0.08	−0.01	0.08	0.01	−0.03
Q2	0.02	−0.02	−0.01	−0.01	−0.01	0.01
Q3	0.06	0.08	−0.02	−0.02	0.13	−0.03
Q4	−0.00	0.09	0.00	−0.05	0.09	−0.00
Q5	−0.01	0.10	0.00	0.04	0.13	−0.03
Q6	0.01	0.13	0.00	−0.00	0.18	−0.02
Q7	0.02	0.10	0.00	0.02	0.11	−0.03
Q8	—	0.00	—	—	−0.03	—
Q9	—	−0.01	—	—	−0.01	—

*Note:* Low longitudinal *κ* coefficients indicate limited agreement between ratings obtained at different surgical stages, which is expected when postoperative morphology changes over time. Because postoperative morphology changes over time, these values should be interpreted as reflecting differences in esthetic classifications between clinical stages rather than reduced reliability of the rating instruments. Comparison of agreement between ratings obtained at different surgical stages (preoperative, 1‐month postoperative, and 6‐month postoperative) using the average of test and retest evaluations. T0 vs. T1 / T1 vs. T2 / T0 vs. T2: Comparisons between preoperative (T0) and postoperative stages at 1 month (T1) and 6 months (T2). Values displayed as 0.00 represent *κ* coefficients with absolute values <0.005 after rounding.

For the categorized VAS, Cohen’s *κ* coefficients ranged from *κ* = − 0.0206 to *κ* = 0.1327. The highest agreement was observed for Q6 between the 1‐month and 6‐month postoperative evaluations (*κ* = 0.1327), whereas the lowest coefficient occurred for Q3 between the preoperative and 6‐month postoperative evaluations (*κ* = − 0.0206). Across all temporal comparisons, most *κ* coefficients remained close to zero.

For the five‐point Likert scale, Cohen’s *κ* coefficients ranged from *κ* = − 0.0454 to *κ* = 0.1765. The highest agreement was observed for Q6 between the 1‐month and 6‐month postoperative evaluations (*κ* = 0.1765), whereas the lowest coefficient occurred for Q4 between the preoperative and 1‐month postoperative evaluations (*κ* = − 0.0454).

Across both rating systems, longitudinal *κ* coefficients remained within the slight agreement category according to the Landis and Koch [29] classification. This pattern was consistent across all morphological items and temporal comparisons, with no comparison reaching moderate agreement.

Negative *κ* coefficients were observed in a limited number of longitudinal comparisons. Given the expected morphological changes occurring between surgical stages, these values should not be interpreted as evidence of inadequate measurement performance but rather as reflecting differences in esthetic classifications across distinct clinical time points.

Detailed Cohen’s *κ* coefficients for each temporal comparison are presented in Table 6.

### 3.5. Exploratory Subgroup Analyses

#### 3.5.1. Evaluator Sex (Supporting Information 3: Table S1)

Exploratory subgroup analyses showed highly comparable esthetic ratings between male and female evaluators across both presentation modes (full‐face and cropped images), both rating systems (categorized VAS and five‐point Likert scale), and all surgical stages.

Minor numerical differences in mean scores were observed in isolated comparisons. For example, using the VAS with full‐face images at the preoperative evaluation, female evaluators assigned slightly higher mean scores to Q1 (2.69) compared to male evaluators (2.60), whereas at the 6‐month postoperative evaluation, male evaluators assigned slightly higher scores to Q9 (4.56) compared to female evaluators (4.37). However, these differences were small in magnitude (generally ≤0.2 points on the 0–10 VAS metric), inconsistent across evaluation sessions, presentation modes, rating scales, and postoperative stages, with no reproducible directional pattern. Overall, postoperative improvements from the preoperative to the 6‐month evaluation followed similar trajectories in both evaluator groups.

Because these subgroup comparisons were purely descriptive and no inferential tests were applied, no *p*‐values or adjustments for multiple comparisons were generated. These analyses should therefore be considered exploratory and hypothesis‐generating rather than confirmatory.

#### 3.5.2. Cleft Index (Supporting Information 4: Table S2)

Similarly, esthetic ratings remained highly comparable between patients classified as Cleft Index 2 and Cleft Index 4. Both groups demonstrated parallel postoperative improvement trajectories across all morphological items, rating scales, and presentation modes.

Although minor numerical differences in mean scores were observed between Cleft Index 2 and Cleft Index 4 groups in selected baseline comparisons, these differences were small in magnitude and were not consistently reproduced across postoperative stages, evaluation sessions, or rating systems. For instance, at the preoperative evaluation using the VAS with full‐face images, patients with Cleft Index 4 received slightly lower mean scores for Q7 (1.95) compared to Cleft Index 2 patients (2.20), consistent with greater baseline morphological severity. However, by the 6‐month postoperative evaluation, mean scores converged substantially (Cleft Index 2: 4.02; Cleft Index 4: 3.85), and similar convergence was observed for most morphological items regardless of cleft severity. As for evaluator sex, these comparisons were descriptive and no inferential tests were performed. Accordingly, the observed numerical differences should be interpreted cautiously and regarded as hypothesis‐generating observations requiring confirmation in larger cohorts.

## 4. Discussion

The present study compared the performance of a five‐point Likert scale, adapted from the anatomical criteria of the Asher–McDade framework, and a 100‐mm VAS for the 3D esthetic assessment of primary unilateral cleft lip repair using a counterbalanced crossover design. Overall, the findings demonstrated moderate‐to‐substantial interscale agreement between the categorized VAS and the five‐point Likert scale across all items, presentation modes, surgical stages, and evaluation sessions. Test–retest agreement was generally lower than interscale agreement and remained within the slight‐to‐fair range, suggesting that image framing influences esthetic perception independently of the rating scale used. As expected, longitudinal agreement between different postoperative stages was limited, reflecting the progressive morphological changes occurring after surgical repair rather than the stability of the measurement instruments. Subgroup analyses according to evaluator sex and cleft index did not reveal consistent or clinically meaningful differences.

### 4.1. Agreement and Validity of the Categorized VAS and the Five‐Point Likert Scale

Agreement between the categorized VAS and the five‐point Likert scale remained consistently within the moderate‐to‐substantial range across all morphological items, surgical stages, presentation modes, and evaluation sessions. These findings indicate that, after categorization of the continuous VAS into five ordinal classes, both instruments generated highly comparable classifications of esthetic outcomes when applied to standardized 3D stereophotogrammetric models evaluated by lay observers.

In addition to the pairwise Cohen’s *κ* analyses, overall inter‐rater reliability was evaluated using the ICC, as recommended for studies involving multiple observers. The ICC analysis demonstrated excellent agreement among the panel of lay observers for both rating systems across all surgical stages. These findings complement, rather than replace, the *κ* analyses, as the two statistics address different methodological questions. Whereas Cohen’s *κ* quantifies agreement between paired ratings (e.g., interscale or test–retest comparisons), the ICC estimates the consistency of ratings across the entire panel of observers. The excellent ICC values therefore provide further support that both the categorized VAS and the five‐point Likert scale can be applied reliably by multiple lay observers in the evaluation of 3D stereophotogrammetric images.

The convergent validity analysis further strengthened these findings. Spearman’s rank correlation coefficients consistently exceeded 0.94 across all presentation modes and surgical stages, demonstrating an excellent monotonic association between the categorized VAS and the five‐point Likert scale. These results indicate that, despite their different measurement properties, both instruments capture highly comparable rankings of postoperative esthetic outcomes. Together with the substantial Cohen’s *κ* values and the excellent ICC estimates, these findings provide complementary evidence supporting the robustness of both rating systems for 3D esthetic assessment.

The present findings extend previous comparisons performed using 2D photographs by demonstrating that a comparable level of agreement can also be achieved within a fully interactive 3D stereophotogrammetric environment. The standardized viewing protocol, which included frontal, lateral, and basal perspectives together with free model rotation, may have contributed to the consistency observed between the two rating systems.

One theoretical methodological advantage of the VAS over an ordinal Likert scale is its continuous measurement structure. Unlike a five‐category ordinal scale, the original 100‐mm VAS allows observers to express esthetic judgments along a continuous spectrum, potentially providing greater measurement resolution and reducing ceiling effects when esthetic outcomes are generally favorable. However, in the present study, the VAS was categorized into five ordinal classes to permit direct agreement analyses with the Likert scale. Consequently, the current kappa analyses evaluate agreement between two ordinal classifications and cannot be interpreted as demonstrating superior sensitivity or discriminatory performance of the original continuous VAS.

Whether the continuous VAS provides additional sensitivity for detecting subtle postoperative esthetic differences remains a plausible theoretical advantage rather than a direct finding of the present study. Demonstrating this potential would require analytical approaches that preserve the continuous nature of the VAS rather than categorization into ordinal classes. Such approaches should be specifically designed to compare continuous and ordinal measurement systems without loss of measurement resolution. Overall, the present findings indicate that both rating systems capture largely overlapping esthetic constructs when applied to standardized 3D facial models. Therefore, both instruments appear suitable for esthetic assessment in this setting, although future investigations preserving the continuous characteristics of the VAS may further clarify whether its higher measurement resolution provides clinically meaningful advantages.

Regarding the isolated negative kappa value (*κ* = −0.06) observed for Q5 at the 6‐month postoperative evaluation, negative kappa coefficients indicate that observed agreement is lower than chance agreement. In our study, these artifactual low or negative values were directly driven by the small clinical sample (*n* = 10 patients) and the resulting highly skewed marginal distributions. Furthermore, this was compounded by potential ceiling effects at the 6‐month follow‐up, where most postoperative ratings concentrated around high esthetic scores, artificially reducing kappa despite high absolute agreement. Because this occurred in only a single comparison among otherwise consistently positive interscale estimates, it should be interpreted as a statistical artifact of low variance rather than evidence of systematic disagreement between the two rating systems.

### 4.2. Test–Retest Agreement

Test–retest agreement was consistently lower than the interscale agreement observed between the categorized VAS and the five‐point Likert scale. Across all morphological items, Cohen’s *κ* coefficients ranged from slight to fair agreement according to the Landis and Koch [29] classification, with the Likert scale generally showing slightly higher *κ* values than the categorized VAS across most evaluation conditions.

The generally low test–retest agreement observed for both rating systems should be interpreted within the context of subjective esthetic assessment rather than as evidence of inadequate measurement performance. Facial attractiveness and postoperative esthetic appearance are perceptual constructs that inherently involve individual judgment, and some degree of within‐observer variability is therefore expected when the same evaluator reassesses identical 3D models after a 2‐week interval. Accordingly, the modest test–retest agreement observed in the present study most likely reflects the intrinsic variability of subjective esthetic perception rather than insufficient reliability of the evaluation protocol itself.

The use of interactive 3D stereophotogrammetric models may have further contributed to this variability. Unlike conventional 2D photographs, observers were able to freely rotate the facial models and inspect multiple anatomical perspectives during each evaluation session. Consequently, subtle differences in viewing sequence or in the anatomical features receiving greater visual attention may have resulted in small variations in repeated ratings despite the use of a standardized evaluation protocol.

In addition, repeated assessments performed ~2 weeks apart may also have been influenced by normal fluctuations in visual attention, observer fatigue, and subtle differences in perceptual focus, all of which are recognized sources of within‐observer variability in subjective esthetic evaluations.

The slightly higher *κ* coefficients observed for the five‐point Likert scale may reflect the inherent characteristics of categorical rating systems, in which evaluators are more likely to assign the same response category during repeated assessments. Conversely, the original continuous structure of the VAS allows finer distinctions in perceived esthetic quality. However, because the VAS was categorized into five ordinal classes to permit direct agreement analyses with the Likert scale, any theoretical advantage associated with its continuous measurement structure was intentionally removed for the purpose of these comparisons. Consequently, the present findings should not be interpreted as evidence supporting the superiority of either rating instrument but rather as demonstrating that both scales produced comparable classifications under the analytical conditions adopted in this study.

Finally, no consistent trend toward higher or lower test–retest agreement was observed across the different postoperative stages. This finding suggests that temporal repeatability remained relatively stable throughout the study period, without evidence of a systematic trend associated with postoperative healing.

### 4.3. Effect of Presentation Mode (FF vs. Non‐FF)

Agreement between cropped versus full‐face images (FF vs. non‐FF) was limited, with kappa values ranging from slight to moderate. This finding indicates that esthetic ratings were influenced by the image presentation mode, despite the standardized protocol requiring the inspection of multiple perspectives.

The influence of perspective on nasolabial evaluation has been previously documented, particularly regarding the diagnostic value of the basal view in assessing nostril symmetry and columellar alignment. The present results confirm that, even within a 3D environment where rotation is possible, the presentation mode affects categorical classification.

These findings highlight the importance of strict standardization of both presentation mode (cropped vs. full‐face) and viewpoint in future 3D esthetic audits. Without explicit orientation protocols, comparisons across centers or studies may be affected by subtle but clinically relevant presentation‐dependent and perspective‐dependent variability.

### 4.4. Longitudinal Agreement Across Surgical Stages

Agreement between ratings obtained at different surgical stages remained consistently low for both the categorized VAS and the five‐point Likert scale. Most Cohen’s *κ* coefficients were close to zero, with occasional negative values, indicating limited agreement between esthetic classifications assigned before surgery and during the postoperative follow‐up.

These findings should not be interpreted as a direct measure of scale responsiveness, since Cohen’s *κ* is an agreement statistic rather than an index of responsiveness or sensitivity to clinical change. Instead, the uniformly low longitudinal agreement is consistent with the expected morphological modifications occurring after primary cleft lip repair. As facial appearance progressively changes between the preoperative, early postoperative, and later postoperative stages, repeated evaluations performed at different time points are expected to generate different esthetic classifications, thereby reducing agreement between stages.

Importantly, the consistently low agreement observed across all temporal comparisons should therefore be interpreted as reflecting genuine differences in postoperative morphology rather than instability or poor reliability of the rating instruments. Both rating systems generated different esthetic classifications across successive clinical stages, consistent with the expected morphological evolution following primary cleft lip repair.

Because the present analyses compared ratings obtained at different clinical stages rather than repeated measurements of an unchanged condition, they should be interpreted as describing longitudinal agreement across postoperative time points rather than the responsiveness of the measurement instruments. Future studies specifically designed to evaluate responsiveness using analytical methods appropriate for continuous longitudinal data will be required to determine whether one scale is more sensitive than the other in detecting clinically meaningful postoperative changes.

### 4.5. Exploratory Subgroup Analyses

Exploratory subgroup analyses according to evaluator sex and cleft severity (Cleft Index) did not reveal consistent differences in esthetic ratings across the evaluated conditions. Although minor numerical differences in mean scores were observed in selected comparisons, these differences were small in magnitude and were not consistently reproduced across presentation modes, rating scales, postoperative stages, or evaluation sessions, and no coherent directional pattern emerged. Given the small magnitude of these numerical differences, their inconsistency across evaluation conditions, and the limited sample size within each subgroup, formal inferential testing was not pursued, as it would have been underpowered and unlikely to yield clinically meaningful conclusions.

These observations suggest that neither evaluator sex nor cleft severity systematically influenced the overall pattern of esthetic assessment within the present dataset. However, these subgroup analyses should be interpreted with caution. Because these subgroup comparisons were purely descriptive and no inferential statistical tests were applied, the observed numerical differences should not be interpreted as evidence of statistically meaningful group differences. Accordingly, these subgroup findings should be regarded as exploratory and hypothesis‐generating rather than confirmatory.

Finally, the relatively small number of patients included in each cleft severity subgroup further limits the interpretation of these analyses. Larger multicenter studies specifically powered for subgroup comparisons will be necessary to determine whether evaluator characteristics or cleft severity meaningfully influence esthetic perception following primary cleft lip repair.

### 4.6. Generalization of Results in the Context of the Literature

The results of the present investigation align with and expand upon established benchmarks in the field of esthetic auditing for cleft lip and palate, confirming that scoring systems for nasolabial appearance remain reliable when transitioned to advanced imaging modalities. The finding of moderate‐to‐substantial inter‐rater agreement for both the Likert and VAS scales is consistent with earlier methodological validations that established the reliability of ordinal and continuous scoring for facial appearance [28]. While traditional protocols were originally developed for 2D photography, the present data demonstrate that these high consensus levels persist within a 3D stereophotogrammetric environment. This suggests that the addition of volumetric depth does not introduce prohibitive noise for lay observers, but instead supports a stable platform for esthetic assessment.

Comparative analyses between continuous and ordinal scales in this study mirror existing evidence suggesting that, while both tools are reliable, continuous measurement systems theoretically provide greater measurement granularity; however, in the present study both instruments demonstrated highly comparable agreement patterns and excellent convergent validity [13]. The substantial interscale concordance observed indicates that both instruments capture a shared “gestalt” or holistic impression of surgical success, a concept frequently supported by large‐scale multicenter esthetic audits [15].

A critical observation from this research pertains to the limited agreement between different cropped versus full‐face images (FF vs. non‐FF), highlighting a significant methodological consideration. This perspective‐dependent variation is corroborated by literature indicating that standardized frontal views often fail to capture the nuances of nasal symmetry and columellar projection, which are only fully revealed through a basal perspective [19]. Consequently, the implementation of strict orientation protocols—including mandatory neck hyperflexion—emerges as a fundamental requirement to ensure that 3D audits remain comparable across different clinical centers [3].

Finally, the uniformly low longitudinal agreement across surgical stages most likely reflects the expected morphological evolution following primary cleft lip repair rather than instability of either rating instrument. Therefore, these coefficients should be interpreted as agreement between evaluations performed at different clinical stages and not as measures of responsiveness. In the context of primary surgical repair, postoperative morphological evolution is instead reflected by the progressive increase in descriptive mean esthetic scores observed from the preoperative stage (T0) to the postoperative evaluations (T1 and T2). Rather than demonstrating scale responsiveness, the uniformly low longitudinal kappa coefficients simply confirm that esthetic classifications differed between clinical stages as a result of primary surgical reconstruction.

### 4.7. Clinical Implications

From a clinical perspective, the results support the continued use of both the Likert scale and the VAS for 3D assessment of primary cleft lip repair outcomes. The substantial inter‐rater agreement indicates that both instruments are suitable for multicenter auditing and group‐level outcome comparisons.

However, the observed viewpoint dependency (frontal, lateral, and basal perspectives) underscores the necessity of standardized orientation protocols in 3D stereophotogrammetry. Additionally, the modest intrarater stability suggests that repeated individual evaluations may be influenced by perceptual variability, reinforcing the importance of multiple raters in outcome research.

The excellent overall inter‐rater reliability demonstrated by the ICC analysis further supports the suitability of both instruments for multicenter outcome assessment.

### 4.8. Limitations

#### 4.8.1. Several Limitations Must Be Acknowledged

The principal limitation of the present study is the relatively small number of patients included. Although only 10 patients were evaluated, the primary objective of this investigation was methodological rather than therapeutic, namely to compare the measurement properties of two esthetic rating systems under highly standardized experimental conditions. Nevertheless, the fully crossed repeated‐measures design generated a large number of paired observations for the reliability analyses, partially compensating for the limited number of clinical cases. This methodological approach is consistent with previous reliability studies evaluating esthetic rating systems in cleft lip and palate, which have similarly relied on repeated‐measures designs rather than conventional power calculations [3, 13, 24–27]. Nevertheless, the limited number of clinical cases restricts the external generalizability of the findings, and the results should therefore be interpreted primarily as methodological and hypothesis‐generating.

The potential effect on generalizability should also be considered carefully. Because all patients were recruited from a single specialized craniofacial center and operated on by a single senior surgeon using a standardized Millard technique, our findings reflect a highly controlled clinical environment. Consequently, these results may not fully generalize to broader multicenter settings where interoperator variability, different surgical protocols, or alternative timing of primary repair are present. Furthermore, although the sample included both left‐ and right‐sided unilateral clefts, it did not encompass the full spectrum of morphological severity commonly encountered in heterogeneous UCLP populations. Larger multicenter studies including more diverse patient cohorts are therefore required to confirm the generalizability of the present findings.

An additional methodological limitation is that direct agreement analyses required categorization of the original continuous VAS into five ordinal classes. Although this harmonization enabled exact comparison with the five‐point Likert scale, it did not preserve the full measurement resolution of the original VAS. Consequently, the present study cannot determine whether retention of the continuous VAS would provide additional discriminatory capability beyond that demonstrated by the harmonized ordinal comparison.

Finally, although unweighted Cohen’s *κ* was selected to quantify exact categorical agreement after harmonization of the two rating systems, future studies specifically designed to preserve the continuous nature of the VAS may benefit from complementary analytical approaches that exploit its full measurement resolution.

### 4.9. Future Perspectives

Future studies should preserve the continuous structure of the VAS when specifically investigating its potential incremental measurement resolution relative to ordinal rating systems. In addition, larger multicenter cohorts and advanced mixed‐effects models may further improve the evaluation of observer variability and longitudinal postoperative changes.

## 5. Conclusions

The present study demonstrates that both the five‐point Likert scale and the VAS provide reliable instruments for the 3D esthetic assessment of primary unilateral cleft lip repair.

Overall inter‐rater reliability was excellent for both rating systems, while pairwise agreement between the categorized VAS and the Likert scale remained consistently moderate to substantial across all evaluation conditions. Furthermore, the two instruments showed excellent convergent validity, indicating that they provide highly comparable rankings of esthetic outcomes despite their different measurement properties.

Test–retest agreement was consistently lower than overall inter‐rater reliability. This contrast highlights a fundamental methodological finding: individual esthetic judgments remain intrinsically variable over time, whereas group‐level assessments by a multirater panel achieve high consistency. Consequently, single‐rater evaluations are insufficient for clinical outcome research. Likewise, agreement between full‐face and cropped image presentations was limited, emphasizing that both the use of multiple evaluators and strict standardization of presentation protocols are essential requirements for consistent 3D esthetic assessment.

Finally, the low agreement observed between different surgical stages most likely reflects the expected morphological evolution following primary cleft lip repair rather than instability of the rating instruments.

Overall, both the five‐point Likert scale and the VAS represent valid tools for 3D esthetic auditing of primary cleft lip repair. Their selection should primarily depend on the intended application, whereas standardized image presentation and the inclusion of multiple evaluators remain essential methodological requirements for future multicenter studies.

**Nomenclature**
VAS: Visual analog scale
UCLP: Unilateral cleft lip and palate
2D: Two‐dimensional
3D: Three‐dimensional.

## Author Contributions

**Mario Zama**: conceptualization, supervision. **Massimo Cordaro**: conceptualization, funding acquisition. **Federica Guglielmi**: data curation, software, visualization. **Antonino Fiorino**: formal analysis, software. **Licia Leccese**: investigation, validation, writing – original draft preparation. **Edoardo Staderini**: methodology, project administration, writing – review and editing. **Carmen Chiara Nacca**: resources, validation, writing – original draft preparation.

## Funding

This research received no external funding.

## Disclosure

All authors have read and approved the final version of the manuscript. All authors have read and agreed to the published version of the manuscript. Federica Guglielmi had full access to all of the data in this study and takes complete responsibility for the integrity of the data and the accuracy of the data analysis.

## Ethics Statement

The study was conducted in accordance with the Declaration of Helsinki, and approved by the Institutional Review Board (or Ethics Committee) of Policlinico Agostino Gemelli—CET Lazio Area 3 (Protocol number 0027003/17 on May 31, 2017).

## Consent

Informed consent was obtained from all parents and legal guardians of the patients about the treatment plan, the participation in the study, and the use of 3D images of their children involved in the study.

## Conflicts of Interest

The authors declare no conflicts of interest.

## Supporting Information

Additional supporting information can be found online in the Supporting Information section.

## Supporting information


**Supporting Information 1** STROBE Statement File—Checklist of items that should be included in reports of observational studies.

**Supporting Information 2** File S2: Esthetic Evaluation Questionnaire—Standardized rating form containing the nine morphological items (Q1–Q9) and scoring guidelines used for the 3D esthetic assessments.

**Supporting Information 3** Table S1. Exploratory subgroup analysis according to evaluator sex: Comparison of mean scores obtained from male (*n* = 25) and female (*n* = 25) lay observers using the Visual Analog Scale (VAS) and the Likert scale. Data are presented for each of the nine assessment items (Q1–Q9) across different viewing conditions (full‐face and non‐full‐face) and time points (T0, T1, T2).

**Supporting Information 4** Table S2. Exploratory subgroup analysis according to cleft severity (Cleft Index): Comparison of esthetic ratings categorized by the severity of the cleft lip and palate (Cleft Index 2 vs. Cleft Index 4). The table reports mean scores for both the Visual Analog Scale (VAS) and the Likert scale across all morphological parameters (Q1–Q9) and experimental time points.

## Data Availability

The datasets generated and/or analyzed during the current study are available from the corresponding author upon reasonable request. The data are not publicity available due to privacy issues.
