# Supplementary material for: Comparison Between the Likert Scale and the Visual Analog Scale for the 3D Esthetic Assessment of Primary Cleft Lip Repair: A Prospective Study
Source: Int J Dent. 2026 Oct 6;2026:3592943. doi: 10.1155/ijod/3592943 (PMC13640046; doi:10.1155/ijod/3592943)
Supplement: Supplementary file 1 [file IJOD-2026-3592943-s001.docx]

**Supplementary Table S1. Exploratory subgroup analysis according to evaluator sex**: Comparison of mean scores obtained from male (n=25) and female (n=25) lay observers using the Visual Analogue Scale (VAS) and the Likert scale. Data are presented for each of the nine assessment items (Q1–Q9) across different viewing conditions (Full-Face and non-Full-Face) and time points (T0, T1, T2).

FF VAS

|  | **MALE** | | | | | | **FEMALE** | | | | | |
| --- | --- | --- | --- | --- | --- | --- | --- | --- | --- | --- | --- | --- |
|  | T-T0 | Re T-T0 | T-T1 | Re T-T1 | T-T2 | Re T-T2 | T-T0 | Re T-T0 | T-T1 | Re T-T1 | T-T2 | Re T-T2 |
| Q1 | 2.60 | 2.41 | 3.55 | 3.32 | 4.31 | 4.04 | 2.69 | 2.91 | 3.68 | 4.03 | 4.43 | 4.64 |
| Q2 | 1.63 | 1.45 | 3.17 | 2.77 | 4.11 | 3.73 | 1.60 | 1.63 | 2.98 | 3.06 | 4.08 | 4.09 |
| Q3 | 1.58 | 1.51 | 3.19 | 2.86 | 3.55 | 3.40 | 1.57 | 1.51 | 2.99 | 3.16 | 3.51 | 3.66 |
| Q4 | 1.74 | 1.65 | 3.37 | 3.08 | 3.74 | 3.45 | 1.59 | 1.59 | 3.1 | 3.22 | 3.53 | 3.61 |
| Q5 | 1.82 | 1.57 | 3.37 | 3.09 | 3.85 | 3.62 | 1.66 | 1.71 | 3.16 | 3.37 | 3.72 | 3.88 |
| Q6 | 1.72 | 1.62 | 3.38 | 3.13 | 3.66 | 3.52 | 1.61 | 1.64 | 3.22 | 3.34 | 3.53 | 3.79 |
| Q7 | 2.24 | 1.95 | 3.46 | 3.29 | 4.01 | 3.75 | 1.96 | 2.09 | 3.4 | 3.64 | 3.89 | 4.12 |
| Q8 | - | - | 2.93 | 2.75 | 4.19 | 3.79 | - | - | 2.89 | 2.91 | 4.14 | 4.20 |
| Q9 | - | - | 3.30 | 2.93 | 4.56 | 4.16 | - | - | 2.95 | 2.83 | 4.37 | 4.42 |

FF LIKERT

|  | **MALE** | | | | | | **FEMALE** | | | | | |
| --- | --- | --- | --- | --- | --- | --- | --- | --- | --- | --- | --- | --- |
|  | T-T0 | Re T-T0 | T-T1 | Re T-T1 | T-T2 | Re T-T2 | T-T0 | Re T-T0 | T-T1 | Re T-T1 | T-T2 | Re T-T2 |
| Q1 | 2.54 | 2.94 | 3.45 | 3.25 | 4.07 | 3.93 | 2.71 | 2.45 | 3.68 | 3.88 | 4.37 | 4.56 |
| Q2 | 1.65 | 1.52 | 3.13 | 2.89 | 3.89 | 3.69 | 1.63 | 1.67 | 3.04 | 3.06 | 3.94 | 4.09 |
| Q3 | 1.62 | 1.61 | 3.09 | 3.00 | 3.40 | 3.31 | 1.61 | 1.67 | 3.02 | 3.19 | 3.47 | 3.61 |
| Q4 | 1.83 | 1.76 | 3.25 | 3.15 | 3.53 | 3.42 | 1.69 | 1.59 | 3.10 | 3.28 | 3.53 | 3.66 |
| Q5 | 1.84 | 1.68 | 3.33 | 3.18 | 3.85 | 3.62 | 1.74 | 1.80 | 3.14 | 3.40 | 3.77 | 3.86 |
| Q6 | 1.80 | 1.69 | 3.27 | 3.23 | 3.51 | 3.48 | 1.68 | 1.77 | 3.26 | 3.39 | 3.53 | 3.75 |
| Q7 | 2.30 | 2.09 | 3.33 | 3.28 | 3.77 | 3.71 | 2.08 | 2.24 | 3.45 | 3.66 | 3.79 | 4.08 |
| Q8 | - | - | 2.90 | 2.85 | 3.95 | 3.74 | - | - | 2.89 | 2.98 | 4.03 | 4.05 |
| Q9 | - | - | 3.27 | 2.99 | 4.37 | 4.13 | - | - | 2.96 | 2.93 | 4.25 | 4.26 |

NO FF VAS

|  | **MALE** | | | | | | **FEMALE** | | | | | |
| --- | --- | --- | --- | --- | --- | --- | --- | --- | --- | --- | --- | --- |
|  | T-T0 | Re T-T0 | T-T1 | Re T-T1 | T-T2 | Re T-T2 | T-T0 | Re T-T0 | T-T1 | Re T-T1 | T-T2 | Re T-T2 |
| Q1 | 2.33 | 2.82 | 3.28 | 3.31 | 4.21 | 3.96 | 2.58 | 2.12 | 3.66 | 3.85 | 4.38 | 4.64 |
| Q2 | 1.32 | 1.3 | 2.91 | 2.8 | 4.04 | 3.71 | 1.36 | 1.49 | 2.84 | 2.98 | 3.97 | 4.16 |
| Q3 | 1.41 | 1.44 | 3.07 | 2.88 | 3.47 | 3.33 | 1.52 | 1.58 | 3.14 | 3.22 | 3.43 | 3.72 |
| Q4 | 1.63 | 1.55 | 3.18 | 3.17 | 3.61 | 3.51 | 1.63 | 1.69 | 3.31 | 3.26 | 3.57 | 3.68 |
| Q5 | 1.63 | 1.58 | 3.26 | 3.12 | 2.53 | 3.64 | 1.73 | 1.74 | 3.34 | 3.48 | 2.61 | 3.93 |
| Q6 | 1.68 | 1.65 | 3.27 | 3.23 | 3.6 | 3.6 | 1.71 | 1.78 | 3.41 | 3.53 | 3.53 | 3.78 |
| Q7 | 2.05 | 1.91 | 3.62 | 3.42 | 4.01 | 3.85 | 2.11 | 2.13 | 3.64 | 3.72 | 4.03 | 4.18 |
| Q8 | - | - | 3.06 | 2.94 | 4.09 | 3.96 | - | - | 3.04 | 3.04 | 4.18 | 4.27 |
| Q9 | - | - | 3.06 | 3.01 | 4.2 | 4.16 | - | - | 3.4 | 2.91 | 4.27 | 4.2 |

NO FF LIKERT

|  | **MALE** | | | | | | **FEMALE** | | | | | |
| --- | --- | --- | --- | --- | --- | --- | --- | --- | --- | --- | --- | --- |
|  | T-T0 | Re T-T0 | T-T1 | Re T-T1 | T-T2 | Re T-T2 | T-T0 | Re T-T0 | T-T1 | Re T-T1 | T-T2 | Re T-T2 |
| Q1 | 2.35 | 2.87 | 3.27 | 3.25 | 4.11 | 3.93 | 2.58 | 2.21 | 3.57 | 3.8 | 4.28 | 4.53 |
| Q2 | 1.41 | 1.4 | 2.93 | 2.89 | 3.87 | 3.65 | 1.49 | 1.54 | 2.89 | 3.02 | 3.84 | 4.05 |
| Q3 | 1.48 | 1.6 | 3.01 | 2.99 | 3.36 | 3.35 | 1.66 | 1.73 | 3.14 | 3.26 | 3.41 | 3.67 |
| Q4 | 1.66 | 1.66 | 3.15 | 3.23 | 3.49 | 3.49 | 1.76 | 1.84 | 3.24 | 3.36 | 3.53 | 3.73 |
| Q5 | 1.7 | 1.69 | 3.22 | 3.22 | 3.49 | 3.63 | 1.83 | 1.89 | 3.3 | 3.51 | 3.74 | 3.95 |
| Q6 | 1.74 | 1.76 | 3.18 | 3.24 | 3.45 | 3.55 | 1.8 | 1.92 | 3.4 | 3.54 | 3.5 | 3.8 |
| Q7 | 2.09 | 2.05 | 3.46 | 3.37 | 3.78 | 3.81 | 2.2 | 2.34 | 3.69 | 3.76 | 3.94 | 4.19 |
| Q8 | - | - | 3.08 | 3.16 | 4.08 | 4.23 | - | - | 2.93 | 3.02 | 3.9 | 3.9 |
| Q9 | - | - | 3.34 | 2.99 | 4.1 | 4.03 | - | - | 3.11 | 3.09 | 4.19 | 4.18 |
