# Supplementary material for: Comparison Between the Likert Scale and the Visual Analog Scale for the 3D Esthetic Assessment of Primary Cleft Lip Repair: A Prospective Study
Source: Int J Dent. 2026 Oct 6;2026:3592943. doi: 10.1155/ijod/3592943 (PMC13640046; doi:10.1155/ijod/3592943)
Supplement: Supplementary file 3 [file IJOD-2026-3592943-s004.docx]

**Supplementary Table S2. Exploratory subgroup analysis according to cleft severity (Cleft Index).**: Comparison of aesthetic ratings categorized by the severity of the cleft lip and palate (Cleft Index 2 vs. Cleft Index 4). The table reports mean scores for both the Visual Analogue Scale (VAS) and the Likert scale across all morphological parameters (Q1–Q9) and experimental time points.

FF VAS

|  | **CLEFT INDEX 2** | | | | | | **CLEFT INDEX 4** | | | | | |
| --- | --- | --- | --- | --- | --- | --- | --- | --- | --- | --- | --- | --- |
|  | T-T0 | Re T-T0 | T-T1 | Re T-T1 | T-T2 | Re T-T2 | T-T0 | Re T-T0 | T-T1 | Re T-T1 | T-T2 | Re T-T2 |
| Q1 | 2.62 | 2.68 | 3.56 | 3.73 | 4.4 | 4.43 | 2.69 | 2.73 | 3.7 | 3.74 | 4.36 | 4.36 |
| Q2 | 1.67 | 1.63 | 3.06 | 2.93 | 4.13 | 4.01 | 1.56 | 1.47 | 3.06 | 2.94 | 4.06 | 3.99 |
| Q3 | 1.64 | 1.5 | 2.96 | 3.05 | 3.58 | 3.48 | 1.5 | 1.52 | 3.18 | 3.03 | 3.48 | 3.53 |
| Q4 | 1.7 | 1.64 | 3.07 | 3.09 | 3.64 | 3.53 | 1.6 | 1.59 | 3.36 | 3.24 | 3.58 | 3.56 |
| Q5 | 1.79 | 1.67 | 3.13 | 3.3 | 3.8 | 3.79 | 1.67 | 1.63 | 3.36 | 3.21 | 3.75 | 3.76 |
| Q6 | 1.7 | 1.61 | 3.1 | 3.24 | 3.61 | 3.72 | 1.61 | 1.66 | 3.47 | 3.26 | 3.56 | 3.64 |
| Q7 | 2.2 | 2.08 | 3.22 | 3.46 | 4.02 | 3.98 | 1.95 | 1.99 | 3.63 | 3.53 | 3.85 | 3.96 |
| Q8 | - | - | 2.79 | 2.83 | 4.25 | 4.03 | - | - | 3.02 | 2.86 | 4.07 | 4.04 |
| Q9 | - | - | 3.08 | 2.9 | 4.5 | 4.33 | - | - | 3.1 | 2.84 | 4.39 | 4.3 |

FF LIKERT

|  | **CLEFT INDEX 2** | | | | | | **CLEFT INDEX 4** | | | | | |
| --- | --- | --- | --- | --- | --- | --- | --- | --- | --- | --- | --- | --- |
|  | T-T0 | Re T-T0 | T-T1 | Re T-T1 | T-T2 | Re T-T2 | T-T0 | Re T-T0 | T-T1 | Re T-T1 | T-T2 | Re T-T2 |
| Q1 | 2.59 | 2.73 | 3.54 | 3.64 | 4.26 | 4.32 | 2.69 | 2.75 | 3.61 | 3.61 | 4.24 | 4.29 |
| Q2 | 1.74 | 1.7 | 3.1 | 3.04 | 3.96 | 4.01 | 1.53 | 1.51 | 3.05 | 2.93 | 3.88 | 3.89 |
| Q3 | 1.67 | 1.64 | 2.96 | 3.1 | 3.46 | 3.48 | 1.56 | 1.64 | 3.14 | 3.12 | 3.42 | 3.49 |
| Q4 | 1.8 | 1.78 | 3.04 | 3.24 | 3.56 | 3.56 | 1.69 | 1.74 | 3.28 | 3.21 | 3.5 | 3.56 |
| Q5 | 1.81 | 1.79 | 3.14 | 3.36 | 3.72 | 3.76 | 1.75 | 1.72 | 3.3 | 3.27 | 3.7 | 3.74 |
| Q6 | 1.77 | 1.73 | 3.14 | 3.27 | 3.55 | 3.72 | 1.68 | 1.75 | 3.39 | 3.37 | 3.5 | 3.56 |
| Q7 | 2.27 | 2.23 | 3.27 | 3.47 | 3.83 | 3.99 | 2.07 | 2.13 | 3.53 | 3.54 | 3.74 | 3.87 |
| Q8 | - | - | 2.87 | 2.93 | 4.05 | 3.96 | - | - | 2.92 | 2.92 | 3.93 | 3.89 |
| Q9 | - | - | 3.08 | 2.97 | 4.35 | 4.24 | - | - | 3.09 | 2.93 | 4.25 | 4.17 |

NO FF VAS

|  | **CLEFT INDEX 2** | | | | | | **CLEFT INDEX 4** | | | | | |
| --- | --- | --- | --- | --- | --- | --- | --- | --- | --- | --- | --- | --- |
|  | T-T0 | Re T-T0 | T-T1 | Re T-T1 | T-T2 | Re T-T2 | T-T0 | Re T-T0 | T-T1 | Re T-T1 | T-T2 | Re T-T2 |
| Q1 | 2.48 | 2.54 | 3.6 | 3.67 | 4.34 | 4.44 | 2.47 | 2.53 | 3.41 | 3.58 | 4.28 | 4.28 |
| Q2 | 1.35 | 1.46 | 2.88 | 3.1 | 4.04 | 4.04 | 1.33 | 1.36 | 2.86 | 2.71 | 3.95 | 3.9 |
| Q3 | 1.64 | 1.61 | 3.18 | 3.27 | 3.47 | 3.61 | 1.31 | 1.42 | 3.04 | 2.89 | 3.42 | 3.5 |
| Q4 | 1.83 | 1.76 | 3.33 | 3.36 | 3.56 | 3.65 | 1.42 | 1.5 | 3.19 | 3.08 | 3.61 | 3.57 |
| Q5 | 1.9 | 1.82 | 3.36 | 3.41 | 2.56 | 2.9 | 1.48 | 1.53 | 3.26 | 3.25 | 2.59 | 2.71 |
| Q6 | 1.95 | 1.9 | 3.41 | 3.51 | 3.57 | 3.77 | 1.45 | 1.56 | 3.3 | 3.3 | 3.55 | 3.64 |
| Q7 | 2.32 | 2.22 | 3.71 | 3.67 | 4.03 | 4.07 | 1.85 | 1.86 | 3.56 | 3.52 | 4.01 | 4.02 |
| Q8 | - | - | 3.13 | 3.04 | 4.22 | 4.21 | - | - | 2.98 | 2.95 | 4.07 | 4.08 |
| Q9 | - | - | 3.19 | 3.06 | 4.33 | 4.28 | - | - | 3.21 | 2.88 | 4.15 | 4.08 |

NO FF LIKERT

|  | **CLEFT INDEX 2** | | | | | | **CLEFT INDEX 4** | | | | | |
| --- | --- | --- | --- | --- | --- | --- | --- | --- | --- | --- | --- | --- |
|  | T-T0 | Re T-T0 | T-T1 | Re T-T1 | T-T2 | Re T-T2 | T-T0 | Re T-T0 | T-T1 | Re T-T1 | T-T2 | Re T-T2 |
| Q1 | 2.47 | 2.59 | 3.52 | 3.61 | 4.25 | 4.33 | 2.5 | 2.61 | 3.38 | 3.54 | 4.17 | 4.24 |
| Q2 | 1.53 | 1.51 | 2.9 | 3.12 | 3.91 | 3.94 | 1.39 | 1.46 | 2.9 | 2.81 | 3.79 | 3.84 |
| Q3 | 1.74 | 1.44 | 3.21 | 3.27 | 3.38 | 3.57 | 1.74 | 1.61 | 2.97 | 3.04 | 3.4 | 3.51 |
| Q4 | 1.89 | 1.85 | 3.29 | 3.42 | 3.52 | 3.67 | 1.55 | 1.68 | 3.12 | 3.19 | 3.51 | 3.59 |
| Q5 | 1.95 | 1.91 | 3.34 | 3.48 | 3.66 | 3.86 | 1.6 | 1.7 | 3.19 | 3.3 | 3.61 | 3.78 |
| Q6 | 1.99 | 2.01 | 3.39 | 3.47 | 3.47 | 3.78 | 1.56 | 1.7 | 3.23 | 3.36 | 3.49 | 3.62 |
| Q7 | 2.34 | 2.4 | 3.68 | 3.65 | 3.85 | 4.08 | 1.97 | 2.04 | 3.51 | 3.55 | 3.9 | 4 |
| Q8 | - | - | 3.09 | 3.16 | 4.06 | 4.16 | - | - | 2.94 | 3.04 | 3.96 | 4.03 |
| Q9 | - | - | 3.21 | 3.11 | 4.25 | 4.21 | - | - | 3.19 | 3.99 | 4.05 | 4.02 |
