# Supplementary material for: Comparison Between the Likert Scale and the Visual Analog Scale for the 3D Esthetic Assessment of Primary Cleft Lip Repair: A Prospective Study
Source: Int J Dent. 2026 Oct 6;2026:3592943. doi: 10.1155/ijod/3592943 (PMC13640046; doi:10.1155/ijod/3592943)
Supplement: Supplementary file 4 [file IJOD-2026-3592943-s003.doc]

STROBE Statement—checklist of items that should be included in reports of observational studies

|  | Item No | Recommendation |
| --- | --- | --- |
| **Title and abstract** | 1 | (*a*) The study is identified as a "Prospective Study" in the title. Pag 1 |
|  |  | (*b*) The abstract outlines the comparison of the Likert scale and VAS, involving 50 observers evaluating 10 children across three time points (T0, T1, T2). It summarizes key findings, including moderate to substantial inter-rater agreement (κ = 0.59 to 0.80) and lower intra-rater reliability. Pag 1 |
| Introduction | | |
| Background/rationale | 2 | The study addresses the subjectivity of surgical outcome assessments in Unilateral Cleft Lip and Palate (UCLP) and the limitations of 2D photography compared to 3D stereophotogrammetry. It highlights the "ceiling effect" and "central tendency bias" of the Likert scale. Pag 2-3 |
| Objectives | 3 | The null hypothesis was that no significant differences would be observed between the 5-point Likert scale and the VAS with respect to agreement, overall inter-rater reliability, convergent validity, and ranking of aesthetic outcomes. Pag 4 |
| Methods | | |
| Study design | 4 | A clinical study with a counterbalanced crossover design, featuring a two-week washout period and scale inversion to minimize recall and order effects. Pag 4 |
| Setting | 5 | Conducted at the Institute of Dentistry of “Fondazione Policlinico Agostino Gemelli” and the Craniofacial Centre of "Bambino Gesù" Children’s Hospital in Rome, Italy. Pag 4 |
| Participants | 6 | (*a*) 10 pediatric patients with non-syndromic UCLP (recruited June–Sept 2017) and 50 lay observers (25 males, 25 females). Exclusion criteria for patients included prior orthopedic or surgical interventions. Pag 4-5 |
| Variables | 7 | Outcomes were defined by nine morphological items (e.g., nasal profile, symmetry, wound healing) assessed via the 5-point Likert scale and a 100-mm continuous VAS. Pag 6 |
| Data sources/ measurement | 8 | 3D facial models were captured using the 3dMDface System. Evaluation was performed using Viewbox 4 software on high-resolution monitors. Pag 5-6 |
| Bias | 9 | Efforts included: crossover design for order/recall bias, blinded raters, standardized orientation sessions to mitigate central tendency bias, and randomized presentation sequence. Pag 7-8 |
| Study size | 10 | Based on a consecutive sample of 10 patients with repeated measures (T0, T1, T2) and 50 evaluators to ensure methodological transparency in reporting reliability. Pag 5 |
| Quantitative variables | 11 | VAS scores were transformed into a 5-category ordinal scale (using specific 20-mm cut-offs) to enable direct comparison with the Likert scale. Pag 5 |
| Statistical methods | 12 | Cohen’s unweighted kappa (κ) for agreement, Spearman’s rank correlation for convergent validity, and independent-samples t-tests for subgroup analyses (sex and cleft index). Pag 8-9 |

Continued on next page

| Results | | |
| --- | --- | --- |
| Participants | 13 | All 50 observers completed the sessions. Analysis followed three stages: potentially eligible, included (10 patients), and analyzed at T0, T1, and T2. Pag 6-7 |
| Descriptive data | 14 | Observer characteristics: 50 laypeople, ages 20–25, 1:1 male-to-female ratio. Patient characteristics: 7 males, 3 females, non-syndromic UCLP. Pag 6-7 |
| Outcome data | 15 | Report of Kappa values for each of the nine items across viewing conditions (FF vs. non-FF) and surgical stages. Pag 10 |
| Main results | 16 | Inter-rater agreement was moderate-to-substantial (κ = 0.54–0.80). Intra-rater reliability was lower (κ = 0.09–0.49). Pag 10-11 |
| Other analyses | 17 | Subgroup analyses by rater sex and cleft index showed no consistent patterns or systematic biases. Pag 16 |
| Discussion | | |
| Key results | 18 | Both the 5-point Likert scale and the categorized VAS demonstrated moderate-to-substantial inter-scale agreement, excellent overall inter-rater reliability, and high convergent validity for 3D aesthetic auditing. Pag 16 |
| Limitations | 19 | Small sample size (10 patients), use of lay observers only, and the possibility of imperfect blinding to treatment stage due to recognizable post-surgical features. Pag 22 |
| Interpretation | 20 | Findings suggest perspective-dependent variation; strict orientation protocols (including basal views) are necessary for clinical research. Pag 18-19-20 |
| Generalisability | 21 | Results indicate that transitioning from 2D to 3D advanced imaging does not compromise consensus levels for facial appearance scoring. Pag 20-21 |
